# Supplementary figures and images for: Participants’ perspectives on a multimodal stress management and comprehensive lifestyle modification program for patients with Crohn’s disease—A qualitative interview study
Source: PLoS One. 2024 Nov 13;19(11):e0313127. doi: 10.1371/journal.pone.0313127 (PMC11559982; doi:10.1371/journal.pone.0313127)

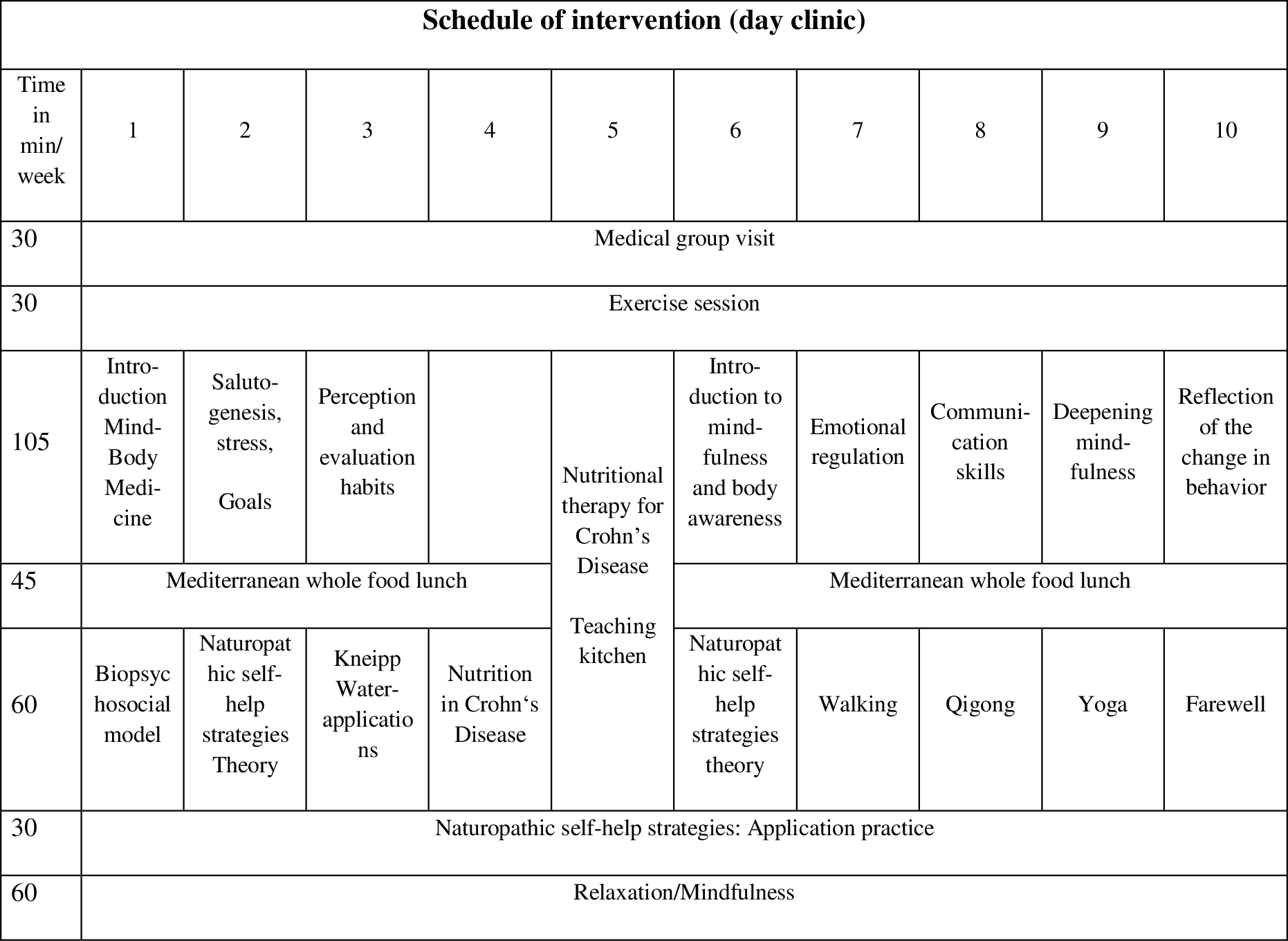

Supplement: S1 Fig — (TIF) [file pone.0313127.s001.tif]
