# Supplementary material for: Participants’ perspectives on a multimodal stress management and comprehensive lifestyle modification program for patients with Crohn’s disease—A qualitative interview study
Source: PLoS One. 2024 Nov 13;19(11):e0313127. doi: 10.1371/journal.pone.0313127 (PMC11559982; doi:10.1371/journal.pone.0313127)
Supplement: Attachment — Submitted filename: Response to Reviewers_Schlee et al.docx [file pone.0313127.s002.docx]

Response to Reviewers for manuscript Schlee et al.

Dear Editor, Dear Reviewers,

Thank you very much for your careful attention to our manuscript and the reviews provided. We appreciated the opportunity to revise again the manuscript and to respond to the reviewers’ comments. We have done our best to address all the comments to the best of our ability and believe that the paper has again benefited from the revisions. We hope that you are satisfied with the changes and find the manuscript appropriate and suitable for publication. Please contact us if you have any questions or concerns.

**Review Comments to the Author**

**Reviewer #1:**

All comments have been addressed.

I am happy with the revisions made to this paper and believe that it is fit to be published in this journal.

**Response from the authors:** Thank you very much.

**Reviewer #2:**

All comments have been addressed.

**Response from the authors:** Thank you very much.

**Reviewer #3:**

This study entitled: Participants’ perspectives on a multimodal stress management and comprehensive lifestyle modification program for patients with Crohn’s Disease – a qualitative interview study is interesting. However, I have some comments.

1. Its better to add a IBD definition at the start of introduction

**Response from the authors:**

Thank you. We added a definition on page 4.

2. "Poor dietary habits and deficiencies in some nutrients are also implicated in the etiology of IBD" add more reference like this reference 10.1002/ptr.7081
**Response from the authors:**

Thank you. We added additional references on page 4.

3. "In addition, it is suggested that the predominant dietary habits, e.g., highly processed foods, sugar consumption, etc., in modern societies can have a negative influence" add more reference like 10.1016/j.clinthera.2020.12.021 and 10.1016/j.dsx.2015.09.015

**Response from the authors:**

Thank you. We added further references on page 4.

4. At the first of the Method section its better to add the design of this study.

**Response from the authors:**

Thank you for your comment. The study design is described at the beginning of the methods section. In order to make the design more comprehensive and provide the overview earlier (at the beginning of the section), we have moved the first reference to Figure 1 there (see page 5).

5. How do you calculate sample size?

**Response from the authors:**

Thank you. We have answered this question during the previous revision and already made changes in the manuscript, see manuscript and previous response. As this was a feasibility and pilot study, sample size was not calculated. We included the sentence on page 7 during the last revision: In this respect no sample size calculation was done because this was a feasibility and pilot study (see page 7).

6. In the method section, its better to add primary and secondary outcomes.

**Response from the authors:**

As this paper based on a qualitative study designed to complement the quantitative investigations and objectives, there were no defined primary and secondary outcomes. To make the research interest clearer, we added the following sentence in the manuscript on page 6: More specifically, the following topics were examined: experience of disease in daily life, reasons for participating in the day clinic program, experiences with the program, perceived changes due to the intervention, and integration of program elements in daily life. Thank you.

7. The quality of figures are so poor and it need to improve.

**Response from the authors:**

Thank you very much for this hint. We have improved the figures.
